# Heart rate variability in HIV-infected and AIDS

**DOI:** 10.1186/cc14662

**Published:** 2015-09-28

**Authors:** Gabriela AM Galdino, Fernanda R Moraes, Gesileny V Silva, Marilita F Accioly

**Affiliations:** 1 Universidade Federal do Triângulo Mineiro, Av. Getúlio Guaritá S/N, Abadia, Uberaba, Minas Gerais, Brazil

## Introduction

The availability of antiretroviral therapy (ART) has resulted in a dramatic reduction in HIV-associated mortality [1]. However, it is also known to induce an array of adverse effects [2-4], as autonomic dysfunction. The use of heart rate variability (HRV), allows the assessment of relative contribution of sympathetic and parasympathetic involvement [1].

## Objective

The objective of this study was to assess the presence of autonomic dysfunction in HIV-infected and acquired immunodeficiency syndrome (AIDS) patients that receive ART. In order to do so, analysis of HRV was applied.

## Methods

A total of 15 subjects with HIV and 34 with AIDS were analyzed. To capture HRV, the heart rate monitor Polar RS810CX was used. The HRV was recorded in a supine position for 20 minutes, and then while performing the respiratory sinus arrhythmia (RSA). The statistical analysis was calculated using ANOVA and Pearson correlation (α = 5%).

## Results

The HIV group had average duration of exposure to the virus of 4.5 ± 3.5 years, and the AIDS group 12.6 ± 6.6 years, both under ART. Only the %REC presented suggestive statistically significant difference (Table 1) of greater parasympathetic modulation in the AIDS group, while the RSA did not show a statistically significant difference (*p *>0.05).

**Table 1:** Time domain analysis.

HRV parameter (ms/%)	HIV	AIDS	*p *value
RMSSD	18.5 (11.5)	23.7 (17)	<0.314
PNN50	3.9 (6.8)	6.9 (9.9)	<0.340
%REC	38.3 (11.2)	32.7 (6.7)	<0.002

Data presented as mean (SD)

## Conclusion

Based on chaos theory, %REC was more sensitive to identify lower parasympathetic modulation in the HIV group, probably due to shorter exposure to antiretroviral therapy. Therefore, it is suggested that the medication used by the population modifies the HRV.
